# Serum Concentration of Antibodies to Mumps, but Not Measles, Rubella, or Varicella, Is Associated with Intake of Dietary Fiber in the NHANES, 1999–2004

**DOI:** 10.3390/nu13030813

**Published:** 2021-03-02

**Authors:** Cynthia B. Van Landingham, Debra R. Keast, Matthew P. Longnecker

**Affiliations:** 1 Ramboll U.S. Consulting, Inc., Monroe, LA 71201, USA; cvanlandingham@ramboll.com; 2 Food & Nutrition Database Research, Inc., Bangor, PA 18013, USA; keastdeb@comcast.net; 3 Ramboll U.S. Consulting, Inc., 3214 Charles B. Root Wynd, Suite 130, Raleigh, NC 27612, USA

**Keywords:** dietary fiber, immunogenicity, antibodies, Mumps, nutrition surveys

## Abstract

Treatment with prebiotics, a type of dietary fiber, was recently shown to increase antibody concentrations following influenza vaccination in a meta-analysis of clinical trials. In observational epidemiologic studies it is not possible to estimate intake of prebiotics, but quantifying intake of dietary fiber is routine. Our objective was to investigate the potential effect of dietary fiber on immunogenicity. We examined serum antibody concentrations (Measles, Mumps, Rubella, and Varicella) in relation to dietary fiber in more than 12,000 subjects in the U.S. National Health and Nutrition Examination Survey (NHANES) for the period 1999–2004. Data from one (1999–2002) or two (2003–2004) dietary recalls were used to calculate fiber intake. For Mumps the adjusted percentage difference in antibody concentration per interquartile range intake in energy-adjusted dietary fiber was 6.34% (95% confidence interval, 3.10, 9.68). Fiber from grain-based foods was more positively associated than fiber from other fiber-containing food groups. The association was slightly larger among subgroups with higher fiber intake, greater interquartile range in fiber intake, and less measurement error. Furthermore, based on the reliability of the diet recalls in 2003–2004, we calculated that the percentage difference per interquartile increment was substantially attenuated by measurement error. Dietary fiber may have a favorable influence on the immunogenicity of some vaccines or natural infections.

## 1. Introduction

Immunocompetence requires adequate intake of protein and micronutrients [1,2,3]. In addition, a potentially important role of certain types of dietary fiber has emerged over the past three decades [4]. Fiber can promote fermentation by the microbiome, producing short-chain fatty acids in the large gut, influencing immune function systemically [5,6]. Prebiotics, a subtype of fiber, are: “a substrate that is selectively utilized by host micro-organisms conferring a health benefit” [7]; most prebiotics are non-digestible oligosaccharides. Treatment with prebiotics to enhance the immunogenicity of vaccines has been investigated, especially for influenza. In a meta-analysis of randomized clinical trials, administration of prebiotic supplements increased antibody titers following influenza vaccination [8]. Estimation of prebiotic intake based on dietary data is presently not possible because nutrient databases for prebiotic content of foods are not sufficiently complete [7]. Thus, observational epidemiologic studies of antibody response in relation to intake of prebiotics are not yet possible. However, because prebiotics are a subtype of dietary fiber, a standard element of nutrient databases, the intake of prebiotics and dietary fiber should be correlated. Among the many remaining questions about the effect of diet on the immune system is whether dietary fiber from typically eaten foods has a beneficial effect on immunogenicity in general.

In a subset of subjects in the National Health and Nutrition Examination Survey [9], the concentration of antibodies to four antigens from natural disease or widely administered vaccines was measured, and a least one 24-h diet recall was administered. Although a single 24-h recall provides an imprecise measure of usual diet and NHANES is a cross-sectional study, these disadvantages were offset by a large sample size. Thus, we were able to test the hypothesis that dietary fiber from typically eaten foods has a beneficial effect on immunogenicity in response to natural infections or vaccines to Measles, Mumps, Rubella, and Varicella.

## 2. Materials and Methods

The NHANES is an ongoing survey of civilian non-institutionalized people residing in the U.S. conducted using a complex design [10]. From 1999–2004, 6- to 49-year-old subjects in NHANES who provided blood specimens had their serum analyzed for the concentration of antibodies to Measles, Rubella, and Varicella; the analysis for Mumps was carried out if sera were available. In the 1999–2000 and 2001–2002 NHANES, one 24-h dietary recall was administered; in 2003–2004 two 24-h dietary recalls were administered, several days apart.

We included participants in our main analyses if they had a measurement of at least one of the antibody titers of interest, data from one 24-h dietary recall if in NHANES 1999–2004 or two 24-h recalls if in NHANES 2003–2004, and complete data for the covariates in the “full model” (see below). 

Antibodies to Measles, Rubella, and Varicella were quantitated using indirect enzyme immunoassays at the California State Department of Health Services, which met the 1988 Clinical Laboratory Improvement Act mandates [11]. The units for Measles and Varicella were optical density and a value ≥ 1 meant the antibody was present. The units for Rubella were international units and a value ≥ 10 meant that the antibody was present. Antibodies to Mumps were quantitated using an enzyme-linked Immunosorbent assay (Wampole Laboratories, Princeton, NJ, USA). The units for Mumps were optical density and a value ≤ 0.9 was negative, 0.901 to 1.099 was equivocal, and ≥ 1.1 was positive; the assay and quality assurance/quality control procedures were conducted at the Centers for Disease Control [9]. 

The dietary survey portion of NHANES consisted of one or two 24-h diet recalls, depending on the year (see above). Based on the consumed amount of individual foods reported in the 24-h recalls, nutrient intake was derived using the NHANES year-specific Food and Nutrient Database for Dietary Studies [12]. Total dietary fiber was the fiber remaining after a food’s digestion by α-amylase, protease, and amyloglucosidase [13]. 

The covariates included in all models were: age, sex, ethnicity, year, and family income to poverty ratio. The additional covariates included in the “full model” were: education, body mass index (BMI), parity, pregnancy and breastfeeding status, smoking, alcohol consumption, dietary intakes (protein, vitamins C and E, carotene, selenium, zinc, B6, folate, magnesium, copper, and vitamin A), and physical activity. Nutrient intake from food and supplements was examined separately. Physical activity in metabolic equivalents of task (MET)∙minutes∙month^−1^ was calculated as described elsewhere [14]. A directed acyclic graph depicting the relations among modeled variables is in Supplementary Materials Figure S1.

The NHANES sampling parameters were used in the statistical procedures so that the results would be generalizable to the U.S. Median and quartiles or percentages were calculated to describe the data. Antibody concentrations were natural log transformed to normalize their distributions. Energy-adjusted fiber intake was calculated. These were calculated by regressing fiber on energy intakes; to the residuals from this model we added the mean intake of unadjusted fiber. An energy-intake-adjustment produces a variable which represents dietary intake from a relative and not absolute perspective [15]. Energy-adjusted intake reflects variation in diet composition that is not confounded by variation in levels of energy intake. One advantage of the “residual approach” is that the interquartile range (IQR) of energy-adjusted intake corresponds more readily to realistic dietary differences than an IQR of non-energy adjusted intake does. To examine how fiber intake varied according to the covariates included in the full model, we calculated the age-adjusted median amount of energy-adjusted fiber according to category of subject characteristics, or the Pearson correlation coefficient of energy-adjusted fiber with the continuous value of the subject characteristic. When calculating correlations with dietary variables, energy-adjusted values of the dietary variables were used. Regression results were expressed as percentage difference in antibody concentration per IQR difference in energy-adjusted fiber intake. First we fitted regression models that adjusted for age, sex, ethnicity, year, and family income to poverty ratio (“minimally adjusted”). Next, we fit models that included the additional covariates (listed above; “full model”). We determined whether addition of a quadratic term for energy-adjusted fiber improved the fit; if it did, we kept the quadratic term in all models for that antibody. We also performed regressions (“full model”) with quartiles of energy-intake-adjusted dietary fiber intake replacing the continuous version of the fiber variable(s).

We examined effect modification by comparing models with and without interaction terms for dietary fiber consumption and age (<12, 12–19, 20+), ethnicity (5 categories), and family income to poverty ratio (tertiles). The comparison was performed using an F-test with *p* < 0.05 indicating a better fit. If the model with the interaction terms provided a better fit to the data, we examined results stratified by the modifying factor. To see if the antibody-fiber association varied by source of fiber we fit models with total fiber replaced by a group of variables representing fiber intake from specific groups of food. The food categories used for this analysis are shown in Supplementary Materials Table S1. 

In sensitivity analyses, we investigated the possibility of bias due to missing data using multiple imputation [16]. Ten versions of the data were created and analyzed, the results were averaged, and adjusted standard errors were calculated. We also reexamined the results for each antibody after excluding subjects who had medical conditions or therapeutic drug use (past 30 days) associated with secondary antibody deficiency [17]. The specific conditions and drugs used to exclude subjects are listed in Supplementary Materials Section S1.

We also estimated the antibody-fiber association that would have been observed in the absence of measurement error in fiber intake, given certain assumptions. This is described in Supplementary Materials Section S2.

The analysis of data was conducted with SAS software, Version 9.4 (SAS Institute, Cary, NC, USA).

## 3. Results

The number of subjects with complete data for Measles, Rubella, and Varicella was 13,225, and for Mumps was 12,616 (Supplementary Materials Figures S2 and S3). The subjects had a median age of 28, and their characteristics were representative of those in the U.S. in the corresponding age range (6–49 years old): the majority were non-Hispanic white, and more than 15% had graduated from college (Table 1; results for Mumps were similar and are presented in Supplementary Materials Table S2). Most females aged 12–49 had children. Most subjects never smoked, and nearly half were light drinkers. The fiber intake distribution was fairly wide and the median of 13 g/day was below the recommended amount—about 30 g/day for those aged 19–30 years [18]. More than a quarter of the subjects aged 12–49 were less active than the current government recommendation of 2000 MET-min/month [14]. The percentages of subjects who had antibody concentrations that were present or positive were: Measles, 95.9%; Mumps, 89.6; Rubella, 91.4; and Varicella, 95.7 (not shown in table). These values were nearly identical to those reported previously for the 1999–2004 NHANES [19,20,21,22]

Among adults the median energy-adjusted fiber intake was higher than among children, and the distribution of fiber intake was wider (Table 2 shows results for those with Measles, Rubella and Varicella data; results for those with Mumps data were similar and are shown in Supplementary Materials Table S3). Females tended to eat more fiber than males. Fiber intake was highest among Mexican Americans and lowest among Non-Hispanic Blacks and those in the “Other race” group. Those with a college degree or the highest income-to-poverty ratio had the highest median fiber intake. Fiber intake increased during the study period. Current smokers had lower fiber intakes. Dietary factors thought to be related to immunocompetence, especially folate and magnesium, were positively correlated with fiber intake. Supplement intake generally showed smaller positive correlations. Fiber intake was lowest among the least active.

**Figure 1:**
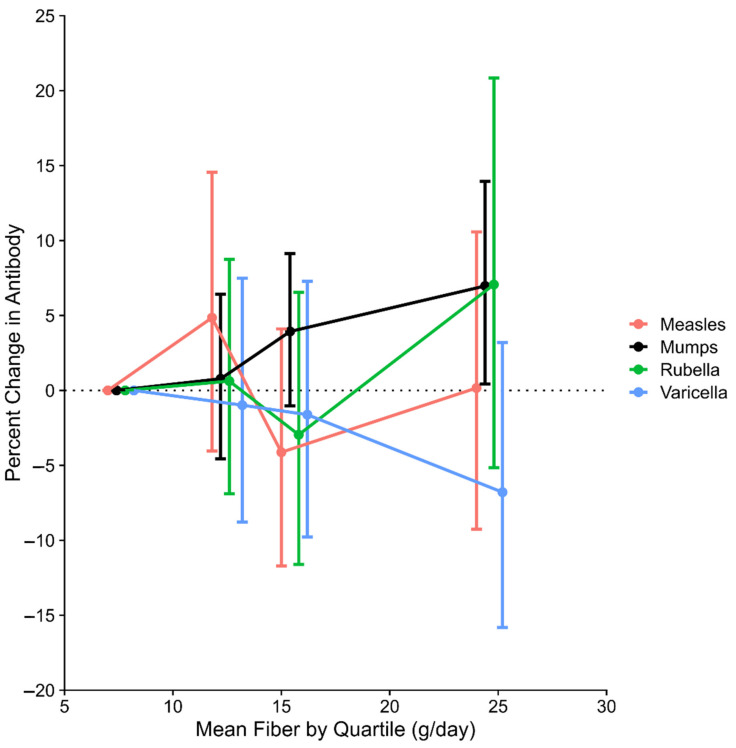
Fully-adjusted percentage change in antibody concentration (and 95% confidence interval) according to quartile of energy-adjusted fiber intake.

The minimally adjusted results indicated a positive association between antibodies to Mumps and Rubella and fiber intake (Table 3), with confidence intervals that were narrower for Mumps. In comparison, the results for Rubella from the fully-adjusted model were much attenuated; for Mumps the adjusted percentage difference (%∆) in antibody concentration per IQR increment in fiber intake was slightly increased, to 6.3% (95% confidence interval [CI], 3.1–9.7). When fiber intake was modeled as quartiles (Figure 1), the percentage increase in antibody concentration for Mumps leveled off at higher intakes, consistent with the improvement of the continuous model fit with the addition of a quadratic term.

For Mumps, the antibody-fiber association varied by age and income-to-poverty ratio (*p* < 0.05). After stratification of results by age and income groups, the association was slightly larger among those aged 12 and older and was much stronger among those in the highest income group (Supplementary Materials Table S4). The fully-adjusted model of Mumps antibody concentration with separate coefficients for fiber from each group of fiber-containing foods fit the data significantly better than the corresponding model with fiber from all sources combined did (Supplementary Materials Table S5). Fiber from grain foods had a more positive association than fiber from other food groups did.

When we used multiple imputation to evaluate the effect of excluding subjects who were missing data on items other than antibody concentrations and fiber, the results for Mumps were slightly attenuated to 5.9%Δ/IQR (95% CI 2.9–9.0), around a 6% decrease (Supplementary Materials Table S6). After exclusion of those with medical conditions or therapeutic drug use associated with secondary antibody deficiency, the results for Mumps were attenuated to 5.2%Δ/IQR (95% CI 0.5–10.0) (Supplementary Materials Table S7), indicating that the fiber association was larger among those with such conditions or drug use. 

Among those in the NHANES 2003–2004, with two dietary recalls available, the overall Pearson correlation coefficient between the first and second 24-h recall energy-adjusted fiber intake was 0.39. Among those < 12 years this was 0.30; for those 12–19 years it was 0.25; and for those 20+ years it was 0.41. By taking the effect of the overall correlation into account, we were able to estimate that due to imprecision, the true percentage difference per interquartile increment in energy-adjusted fiber intake was 140% or more greater than that shown for Mumps in Table 3 (See Section S2 of the Supplementary Materials). 

## 4. Discussion

In this large cross-sectional study, representative of the U.S. population aged 6–49 years in 1999–2004, those who ate more dietary fiber had higher concentrations of antibodies to Mumps, but this was not the case for Measles, Rubella, or Varicella. The size of the association with Mumps was modest.

Routine use of the Mumps vaccination at 15 months of age began in 1977, and routine administration of a second dose at ages 4–6 years began in 1989 [20]. Thus, among those in our study, the 6–11 age group should have received two Mumps vaccinations, most of the 12–19 group should have received one, and most of those aged 20+ y should have had natural infections. Routine vaccination began against Measles in 1963, Rubella in 1969, and Varicella in 1995 [22,23]. After vaccination, the initial antibody response and rate of decay is more predictable for Mumps compared with Measles and Rubella [24]; whether this holds for natural infections is unclear. Nonetheless, higher predictability would improve our ability to detect an effect of fiber on the antibody concentration. 

Modification of antibody response by fiber could vary according to type of vaccine or natural infection, but to our knowledge no other data address this. Studies of prebiotics—a type of dietary fiber—provide some relevant information. The effect of prebiotics on the response to the vaccine for influenza has been studied more than for other vaccines, with 212 clinical trial subjects included in a recent meta-analysis [8]; the results for antibodies to influenza antigen A/H1N1 were heterogeneous but most studies showed an increase of roughly 30–50%. The other two antibodies examined in the meta-analysis, to other strains of influenza, also had higher antibody concentrations with prebiotic use, but the results were not statistically significant. Trials of prebiotics among infants have shown no benefit for a variety of vaccine-induced antibodies [25,26,27,28]. Other relevant data are discussed in Supplementary Materials Section S3. Among the reports we identified, none examined antibodies to Mumps. 

We found that the association of Mumps antibody concentrations with fiber was slightly larger among those aged 12 or more years. The smaller association among younger subjects could have been due to a combination of less variation in fiber intake (Table 2) and more measurement error in diet, or because fiber affects the rate of antibody decay, making effects more evident at older ages. The positive findings among those aged 12–19 y suggests that vaccine response as well as response to natural infection were affected. The antibody-fiber relation for Mumps was also stronger among those with higher income. The high-income group had higher fiber intake and a wider distribution of fiber intake, and both would increase statistical power to detect an association.

Given that day-to-day variation in diet exists, because our assessment of diet was based on one or two 24-h diet recalls, we had an imprecise measure of usual intake for each subject. Imprecision in the fiber measure means that observed associations with antibody concentrations were attenuated. We estimated the effect of imprecision on the association for Mumps by taking into account the correlation between two energy-adjusted fiber intakes available in NHANES 2003–2004; this suggested that the estimates for the fiber “effect” would have been more than 140% greater in the absence of imprecision (Supplementary Materials Section S2). The imprecision also applied to the dietary confounders, meaning that the fiber “effect” estimate might have been influenced by residual confounding. In these data, however, none of the dietary factors other than fiber was related to the Mumps antibody concentration, possibly because this was generally a well-nourished population. Another uncertainty arose from the inaccuracy in 24-h diet recalls [29], which would have added to the imprecision and could have biased the results. Furthermore, if prebiotics in fiber increased Mumps antibodies, use of fiber as an exposure measure would have attenuated the findings. Comprehensive nutrient databases for prebiotic content of foods do not exist at present. The broad soluble/insoluble fiber dichotomy once used has been declared obsolete by the National Academy of Sciences, thus this breakdown has not been implemented in USDA food composition databases used by NHANES to determine nutrient intake [30].

Our data had other important weaknesses. Natural infection induces a higher concentration of antibodies than vaccination does [22,31]. Antibody concentrations at one point in time reflect initial response, rate of decay, time since vaccination or infection, and probably other factors such as past diet, age, and recent infections or vaccinations with other antigens. The influence of these factors may vary by vaccine or infection, by individual, and differently for individuals according to vaccine [24]. We did not know if the antibodies measured were from natural infection or vaccination. We did not know when the infection or last vaccination occurred. The assay used for Varicella gave a substantial proportion of false negatives [22]. Although we considered age and other factors in our analysis, we were unable to account for many other sources of variation in antibody concentration, meaning our outcome measure as an indicator of immune response was imprecise. This imprecision decreased our statistical power to detect an association. Despite the limitations incurred by use of NHANES data, we were able to detect a modest association for Mumps, possibly because of the large sample size. Nonetheless, confirmation of the Mumps antibody-fiber association in large studies with a comprehensive diet assessment and data analysis would increase confidence. 

Dietary fiber affects the production of short-chain fatty acids (SCFAs) in the large bowel, which are absorbed systemically and influence the immune system [6]. The immune effects of SCFAs include decreased inflammation and autoimmunity, and there may also be effects on parenteral vaccine response [32,33]. Antibiotics administered at the time of an influenza vaccine caused a reduced A/H1N1 antibody response [34]. The role of SCFAs in the B-cell response has been studied in the acute setting, rather than what may be more relevant to our findings—effects on long-lived plasma cells that produce antibodies [35]. Knowledge about the effects of dietary microbiota-accessible carbohydrate on human health, despite decades of research, is at an early stage [36]. The association that we found, however, could be due to chance, or to some other effect of fiber. For example, if fiber decreased systemic concentrations of immunotoxicants, this could increase antibody concentrations [37,38]. 

Higher concentration of Mumps antibodies may provide greater protection against infection, but unlike Measles and Rubella, an antibody concentration that defines immunity has not been established [20]. Nonetheless, reduced T cell-dependent antibody response (TDAR) is considered evidence of immunotoxicity and is measured by antibody response following exposure to a specific antigen [39]. 

Vaccine response has been used as a critical endpoint in at least one risk assessment, for perfluoroalkyl substances (PFASs), a ubiquitous environmental contaminant [40]. Serum concentrations of PFASs have been associated with lower antibody concentrations in humans [38], though the results vary by antibody and are most consistent for Tetanus and Diphtheria [41]. Stein et al. [42], using a subset of NHANES data from 1999–2004, found that inverse associations of PFAS serum concentrations with Mumps antibody titers, but not those of Measles, Rubella, or Varicella, achieved statistical significance. We and others recently reported that those with more fiber in their diets tend to have lower PFAS serum concentrations [37,43]. One implication of our results is that the Mumps antibody-PFAS association in NHANES may be due to confounding by dietary fiber. Assessing the degree to which this association is confounded by fiber will be challenging given the difficulty of measuring diet well. 

Antibodies were measured in this study following viral infections or vaccination with live attenuated viruses [24]. Our results leave open the possibility that an association between fiber and other antibodies may be present, but at this point we have little indication which ones other than influenza would most likely be similarly affected.

## Figures and Tables

**Table 1:** Characteristics of National Health and Nutrition Examination Survey (NHANES) subjects (1999–2004) included in the main analyses ^a^.

Characteristic	Median (and Quartiles), or Percent (*n =* 13225)
Age	28 (17, 39)
Sex	
Female	48.2
Male	51.8
Race/Ethnicity	
Mexican American	10.1
Other Hispanic	5.9
Non-Hispanic White	66.6
Non-Hispanic Black	12.1
Other Race	5.3
Education	
<9th grade	16.6
Grades 9 to 11	16.6
High School or GED (includes those in Grade 12)	21.2
Some College	26.1
College	19.5
Income-Poverty Ratio	2.6 (1.3, 4.5)
Survey Year	
1999–2000	29.9
2001–2002	35.9
2003–2004	34.2
BMI (kg/m^2^)	24.9 (21.0, 29.6)
Parity (females, ages 12–49) ^b^	
0 children	40.7
1 child	16.1
2 or more	43.2
Pregnant (females, ages 12–49) ^b^	4.8
Breastfeeding (females, ages 12–49) ^b^	2.6
Smoking (ages 12–49) ^c^	
Never [<100 lifetime cigarettes]	57.3
Former [not current smoker]	17.6
Smoker [<1 pack per day]	14.8
Heavy Smoker [≥1 pack per day]	10.3
Alcohol Use (ages 20–49) ^d^	
Never [<12 lifetime drinks]	12.0
Former [0 drinks last 12 months]	2.0
Light Drinker [<1 drink per week]	46.4
Drinker [<7 drinks per week]	36.6
Heavy Drinker [≥7 drinks per week]	3.0
Dietary Intake	
Crude Dietary Fiber (g/day)	13.3 (8.9, 19.4)
Energy Adjusted Fiber (g/day)	13.8 (10.6, 18.3)
Energy Adjusted Fiber (g/day)/IUR	1.8 (1.4, 2.4)
Total Energy Intake (kcal/day)	2169 (1634, 2854)
Vitamin C (mg)	60.6 (27.6, 124.0)
Vitamin E (mg)	6.3 (4.2, 9.4)
Carotene (mcg RE)	784 (335, 2332)
Protein (gm)	77.6 (55.7, 104.8)
Selenium (mcg)	99.1 (70.5, 138.0)
Zinc (mg)	10.9 (7.5, 15.8)
Vitamin B6 (mg)	1.7 (1.1, 2.4)
Folate (mcg)	361.5 (248.0, 517.5)
Magnesium (mg)	252.0 (179.9, 345.0)
Copper (mg)	1.1 (0.8, 1.6)
Vitamin A (mcg)	526.9 (294.0, 868.2)
Supplements	
Crude Supplement Fiber (g/day)	0.0 (0.0, 0.0)
Vitamin C (mg)	0.0 (0.0, 49.7)
Vitamin E (mg)	0.0 (0.0, 7.2)
Carotene (mg)	0.0 (0.0, 0.0)
Protein (gm)	0.0 (0.0, 0.0)
Selenium (mcg)	0.0 (0.0, 0.0)
Zinc (mg)	0.0 (0.0, 0.5)
Vitamin B6 (mg)	0.0 (0.0, 0.8)
Folate (mcg)	0.0 (0.0, 66.7)
Magnesium (mg)	0.0 (0.0, 0.0)
Copper (mg)	0.0 (0.0, 0.0)
Vitamin A (mcg)	0.0 (0.0, 197.9)
Met-Min/Month (ages 12–49) ^e^	
<2000	27.9
2000–3999	17.8
4000–5999	11.3
6000–7999	9.0
8000+	33.9
Antibody concentration (untransformed)	
Measles	8.6 (4.2, 14.6)
Mumps	2.6 (1.7, 3.7) ^f^
Rubella	46.8 (22.4, 85.5)
Varicella	13.3 (7.9, 18.4)

^a^ Values shown are for those with measurements of Measles, Rubella, and Varicella. See footnote f below for information about results for Mumps. ^b^ Females 12–49, *n =* 5476. ^c^ For smoking data, *n =* 9531. ^d^ For alcohol data, *n =* 4891. ^e^ For Met-Min/Month data, *n =* 7982. ^f^ For Mumps, the *n =* 12,616; characteristics of the 12,616 are in Supplementary Materials Table S2; they are similar to those above.

**Table 2:** Age-adjusted median amount of energy-adjusted dietary fiber (g/day) according to category of subject characteristic (and quartiles) or Pearson correlation coefficient of energy-adjusted dietary fiber with continuous value of the subject characteristic.

Characteristic	Median or r (*n =* 13225) ^a^
Age	
6 to < 12 years	13.7 (11.4, 16.4)
12 to < 20 years	12.7 (10.2, 16.3)
20–49 years	14.3 (10.6, 19.2)
Sex	
Female	14.2 (11.3, 18.2)
Male	13.4 (10.0, 18.4)
Race/Ethnicity	
Mexican American	16.1 (12.3, 21.4)
Other Hispanic	14.1 (11.3, 18.4)
Non-Hispanic White	14.0 (10.6, 18.4)
Non-Hispanic Black	12.2 (9.6, 15.4)
Other Race	12.8 (9.8, 17.3)
Education	
<9th grade	13.7 (11.2, 16.9)
Grades 9 to 11	12.5 (9.4, 16.6)
High School or GED (includes those in Grade 12)	12.8 (9.5, 17.0)
Some College	13.9 (10.6, 18.6)
College	16.7 (12.7, 23.0)
Income-Poverty Ratio	
1st Tertile	13.2 (10.1, 17.2)
2nd Tertile	13.5 (10.3, 17.6)
3rd Tertile	14.8 (11.4, 19.9)
Survey Year	
1999–2000	13.2 (9.9, 18.1)
2001–2002	13.9 (10.6, 18.6)
2003–2004	14.2 (11.3, 18.1)
BMI (kg/m^2^)	−0.05
Parity (females, ages 12–49) ^b^	
0 children	14.4 (11.4, 18.7)
1 child	13.9 (10.9, 18.3)
2 or more	14.2 (11.2, 18.8)
Pregnant (females, ages 12–49) ^b^	
No	14.2 (11.2, 18.5)
Yes	15.0 (11.6, 19.7)
Breastfeeding (females, ages 12–49) ^b^	
No	14.2 (11.2, 18.5)
Yes	15.6 (12.1, 21.6)
Smoking (ages 12–49) ^c^	
Never [<100 lifetime cigarettes]	14.9 (11.2, 19.7)
Former [not current smoker]	15.0 (11.6, 20.7)
Smoker [< 1 pack per day]	12.0 (9.0, 15.8)
Heavy Smoker [≥ 1 pack per day]	10.8 (8.0, 14.4)
Alcohol Use (ages 20–49) ^d^	
Never [<12 lifetime drinks]	15.4 (11.9, 20.8)
Former [0 drinks last 12 months]	15.6 (9.9, 20.0)
Light Drinker [<1 drink per week]	14.2 (10.7, 19.2)
Drinker [<7 drinks per week]	13.9 (9.9, 19.1)
Heavy Drinker [≥7 drinks per week]	13.5 (10.3, 19.8)
Dietary Intake	
Vitamin C (mg)	0.22
Vitamin E (mg)	0.27
Carotene (mcg RE)	0.26
Protein (gm)	0.08
Selenium (mcg)	0.07
Zinc (mg)	0.15
Vitamin B6 (mg)	0.29
Folate (mcg)	0.44
Magnesium (mg)	0.67
Copper (mg)	0.36
Vitamin A (mcg)	0.18
Supplements	
Crude Supplement Fiber (g/day)	0.01
Vitamin C (mg)	0.01
Vitamin E (mg)	−0.00
Carotene (mg)	0.04
Protein (gm)	0.05
Selenium (mcg)	0.05
Zinc (mg)	0.07
Vitamin B6 (mg)	0.04
Folate (mcg)	0.08
Magnesium (mg)	0.06
Copper (mg)	0.07
Vitamin A (mcg)	0.00
Met-Min/Month (ages 12–49) ^e^	
<2000	13.6 (10.3, 18.5)
2000–3999	15.0 (11.0, 19.3)
4000–5999	14.5 (11.4, 19.9)
6000–7999	14.3 (10.8, 19.3)
8000+	14.4 (10.7, 19.3)

^a^ Results shown are for the 13,225 subjects with data on Measles, Rubella, and Varicella. Results for Mumps (*n =* 12616) were similar, and can be found in Supplementary Materials Table S2. ^b^ Females aged 12–49, *n =* 5476. ^c^ For smoking data, *n =* 9531. ^d^ For alcohol data, *n =* 4891. ^e^ For Met-Min/Month data, *n =* 7982.

**Table 3:** Percent difference in antibody concentration (%∆) per interquartile range increment in dietary fiber intake (and 95% confidence interval).

	Minimally-Adjusted		Full Model	
Antibody Type	%∆	(95% Cl)	%∆	(95% Cl)
Measles	2.42	(−1.59, 6.59)	0.47	(−4.34, 5.51)
Mumps *	5.36	(2.17, 8.64)	6.34	(3.10, 9.68)
Rubella	4.04	(0.60, 7.61)	0.98	(−4.19, 6.43)
Varicella	−2.98	(−5.97, 0.11)	−2.64	(−5.99, 0.83)

* Fiber results based on a quadratic representation.

## Data Availability

The link to the publicly archival NHANES datasets is: NHANES III (1988–1994)—Data Files (cdc.gov).

## Supplementary Materials

The following are available online at https://www.mdpi.com/2072-6643/13/3/813/s1. Section S1: Definition of immunocompromising conditions or medications; Section S2: Estimation of the effect of measurement error; Section S3: A discussion of other relevant data on prebiotics and immunogenicity; Figure S1: Directed acyclic graph showing relations among variables; Figure S2: Reasons for exclusion from the main analysis (Measles, Rubella, Varicella); Figure S3: Reasons for exclusion from the main analysis (Mumps); Table S1: Fiber-containing food groups used in the data analysis; Table S2: Characteristics of subjects in the Mumps analysis; Table S3: Relation of fiber intake to subject characteristics, Mumps analysis; Table S4: Mumps antibody-fiber association among selected subgroups; Table S5: Food-group specific fiber associations; Table S6: Multiple imputation results; Table S7: Results after exclusion of immunocompromised subjects.

## Abbreviations

IQR	interquartile range
MET	metabolic equivalents of task
NHANES	National Health and Nutrition Examination Survey
%∆	percentage difference
SFCA	short-chain fatty acids
TDAR	T cell-dependent antibody response
