# Supplementary material for: Serum Concentration of Antibodies to Mumps, but Not Measles, Rubella, or Varicella, Is Associated with Intake of Dietary Fiber in the NHANES, 1999–2004
Source: Nutrients. 2021 Mar 2;13(3):813. doi: 10.3390/nu13030813 (PMC8001807; doi:10.3390/nu13030813)
Supplement: Supplementary file 1 [file nutrients-13-00813-s001.pdf]

Supplementary Materials (SM)

Serum concentration of antibody to Mumps, but not Measles, Rubella, or Varicella, is associated with intake of dietary fiber in the NHANES, 1999-2004

Cynthia B. Van Landingham, Debra R. Keast, Matthew P. Longnecker

Table of Contents

Item and brief description	Page
SM Section 1: Definition of immunocompromising conditions or medications	2
SM Section 2: Estimation of the effect of measurement error	3
SM Section 3: A discussion of other relevant data on prebiotics and immunogenicity	5
SM Figure 1: Directed acyclic graph showing relations among variables	6
SM Figure 2: Reasons for exclusion from the main analysis (Measles, Rubella, Varicella)	7
SM Figure 3: Reasons for exclusion from the main analysis (Mumps)	8
SM Table 1: Fiber-containing food groups used in the data analysis	9
SM Table 2: Characteristics of subjects in the Mumps analysis	12
SM Table 3: Relation of fiber intake to subject characteristics, Mumps analysis	15
SM Table 4: Mumps antibody-fiber association among selected subgroups	18
SM Table 5: Food-group specific fiber associations	19
SM Table 6: Multiple imputation results	20
SM Table 7: Results after exclusion of immunocompromised subjects	21

SM Section 1: Definition of immunocompromising conditions or medications

Following Patel et al. (2019), we excluded people who reported ever having the conditions listed below or who were taking the medications listed below in the past 30 days.

Conditions:

- Blood cancer
- Leukemia
- Lymphoma
- Renal failure or compromise
- HIV

Medications:

- Adrenal corticosteroids
- Oncolytics
- Antineoplastics
- Antimetabolites
- Azathioprine
- Bortezomib
- Carbamazepine
- Chlorambucil
- Chlorpromazine
- Clozapine
- Dasatinib
- Gold
- Ibrutinib
- Imatinib
- Lamotrigine
- Mercaptopurine
- Mycophenolate mofetil
- Penicillamine
- Phenytoin
- Rituximab
- Sulphasalazine
- Valproic acid

SM Section 2: Estimation of the Mumps antibody-fiber association in the absence of measurement error

As noted in the main report, use of one or two 24-hour dietary recalls provided an imprecise measure of usual fiber intake, due to the day-to-day variation in diet. Because we had two 24-hour dietary recalls for each subject in the 2003-2004 NHANES wave, we were able to estimate what the Mumps antibody-fiber association would have been in the absence of this imprecision.

We could de-attenuate the initial estimate of β_{fiber} based on the fiber intake in one recall but not if we had used the average fiber based on two recalls. So, we fit the models of Mumps using the one 24-hour recall data for subjects from the 1999-2002 waves and the first 24-hour recall data from 2003-2004. We then estimated $\beta_{fiber\text{-}DA}$ (the de-attenuated estimate) using β_{fiber} as follows (Keogh et al. 2020):

$$\beta_{fiber\text{-}DA} = \beta_{fiber}/r$$

Where r is the Pearson correlation coefficient between the energy-adjusted fiber values of the two 24-hour recalls. Here we are treating the two 24-hour recalls as a replicates study. Because we have a large sample size for the replicates study (n=4,148), we assumed no additional variance in the estimate of $\beta_{fiber\text{-}DA}$ due to the de-attenuation (Fibrinogen Studies Collaboration, 2009), and assumed $\beta_{fiber\text{-}DA}$ had the same t as β_{fiber}. We then repeated this procedure using the second 24-hour recall data for the 2003-2004 subjects (and the single day data for the 1999-2002 subjects), and calculated the inverse variance mean of the two results.

β_{fiber} (and 95% CI) and corresponding % difference in Mumps antibody (and 95% CI) obtained using one 24-hour diet recall*

	β_{fiber}	%Δ in Mumps antibody
Mumps antibody	0.0557 (0.0217, 0.0897)	5.73 (2.20, 9.38)

*The model of Mumps used was otherwise the same as the "full" model in the main report (n=12,616); weighted mean results from the use of the first or second recalls for the 2003-2004 subjects (plus the 1999-2002 subjects) are shown.

Pearson correlation coefficients between energy-adjusted dietary fiber from the two diet recalls (n=4,148)

No transformation of energy-adjusted fiber	Box Cox transformation of energy-adjusted fiber
0.40	0.39

De-attenuated estimates of % difference in Mumps antibody (and 95% CI), with comparison to values based on one or two 24-hour recalls

	%Δ in Mumps antibody$_{fiber\text{-}DA}$	%Δ in PFAS$_{fiber\text{-}1\ or\ 2\ 24\text{-}h}$*	% increase
Mumps antibody	15.35 (5.73, 25.84)	6.34	142

* These are the same results shown for the final model in Table 3.

The IQD in energy-adjusted fiber intake for the de-attenuated estimates is 6.0 g/d, as compared with the observed value of 7.7 for the IQD based on one or the average of two 24-hour diet recalls. The IQD for the de-attenuated energy-adjusted fiber intake was calculated with the NRC method (Shaw et al., 2020), using Box Cox transformed values and the distribution of energy-adjusted fiber in the first 24-hour recall. The difference between the de-attenuated and observed IQDs means that the % increase shown above is a slight underestimate.

References

Fibrinogen Studies Collaboration. Correcting for multivariate measurement error by regression calibration in meta-analyses of epidemiological studies. Stat Med. 2009 Mar 30;28(7):1067-92.

Keogh RH, Shaw PA, Gustafson P, Carroll RJ, Deffner V, Dodd KW, Küchenhoff H, Tooze JA, Wallace MP, Kipnis V, Freedman LS. STRATOS guidance document on measurement error and misclassification of variables in observational epidemiology: Part 1-Basic theory and simple methods of adjustment. Stat Med. 2020 Jul 20;39(16):2197-2231.

Shaw PA, Gustafson P, Carroll RJ, Deffner V, Dodd KW, Keogh RH, Kipnis V, Tooze JA, Wallace MP, Küchenhoff H, Freedman LS. STRATOS guidance document on measurement error and misclassification of variables in observational epidemiology: Part 2-More complex methods of adjustment and advanced topics. Stat Med. 2020 Jul 20;39(16):2232-2263.

SM Section 3: A discussion of other relevant data on prebiotics and immunogenicity

Response to vaccination for pneumococcus was examined in a small randomized clinical trial of prebiotics in older adults and showed no effect (Bunout et al., 2002). Human breast milk contains microbiota-accessible carbohydrate and has a beneficial effect on the developing immune system (Pretorius et al., 2018). Its effect on vaccine response in observational studies, however, has been mixed for Hemophilus influenza b (Decker et al., 1992; Greenberg et al., 1994; Pabst and Spady 1990; Scheifele et al. 1992; Silverdal et al. 2007); for antibody to pneumococcal serotype 14 antigen one study supported a transient benefit (Silverdal et al., 2007); and for other antibodies the data do not support an increase in concentration (Deforest et al., 1973; John et al., 1976; Pabst et al., 1997; Rennels, 1996).

Bunout D, Hirsch S, Pía de la Maza M, Muñoz C, Haschke F, Steenhout P, et al. Effects of prebiotics on the immune response to vaccination in the elderly. JPEN J Parenter Enteral Nutr. 2002 Dec;26(6):372–6.

Decker MD, Edwards KM, Bradley R, Palmer P. Comparative trial in infants of four conjugate Haemophilus influenzae type b vaccines. J Pediatr. 1992 Feb;120(2 Pt 1):184–9.

Deforest A, Parker PB, DiLiberti JH, Yates HT, Sibinga MS, Smith DS. The effect of breast-feeding on the antibody response of infants to trivalent oral poliovirus vaccine. J Pediatr. 1973 Jul;83(1):93–5.

Greenberg DP, Vadheim CM, Partridge S, Chang SJ, Chiu CY, Ward JI. Immunogenicity of Haemophilus influenzae type b tetanus toxoid conjugate vaccine in young infants. The Kaiser-UCLA Vaccine Study Group. J Infect Dis. 1994 Jul;170(1):76–81.

John TJ, Devarajan LV, Luther L, Vijayarathnam P. Effect of breast-feeding on seroresponse of infants to oral poliovirus vaccination. Pediatrics. 1976 Jan;57(1):47–53.

Pabst HF, Spady DW, Pilarski LM, Carson MM, Beeler JA, Krezolek MP. Differential modulation of the immune response by breast- or formula-feeding of infants. Acta Paediatr. 1997 Dec;86(12):1291–7.

Pabst HF, Spady DW. Effect of breast-feeding on antibody response to conjugate vaccine. Lancet. 1990 Aug 4;336(8710):269–70.

Pretorius R, Prescott SL, Palmer DJ. Taking a prebiotic approach to early immunomodulation for allergy prevention. Expert Rev Clin Immunol. 2018;14(1):43–51.

Rennels MB. Influence of breast-feeding and oral poliovirus vaccine on the immunogenicity and efficacy of rotavirus vaccines. J Infect Dis. 1996 Sep;174 Suppl 1:S107-111.

Scheifele D, Bjornson GJ, Guasparini R, Friesen B, Meekison W. Breastfeeding and antibody responses to routine vaccination in infants. Lancet. 1992 Dec 5;340(8832):1406.

Silfverdal SA, Ekholm L, Bodin L. Breastfeeding enhances the antibody response to Hib and Pneumococcal serotype 6B and 14 after vaccination with conjugate vaccines. Vaccine. 2007 Feb 9;25(8):1497–502.

OR Figure 1: Directed acyclic graph showing relations among variables

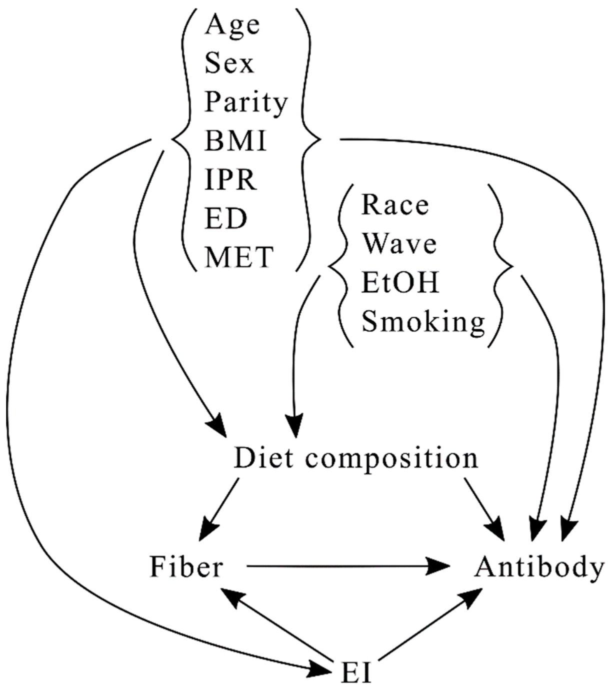

SM Figure 2: Reasons for exclusion from the main analysis (Measles, Rubella, Varicella)

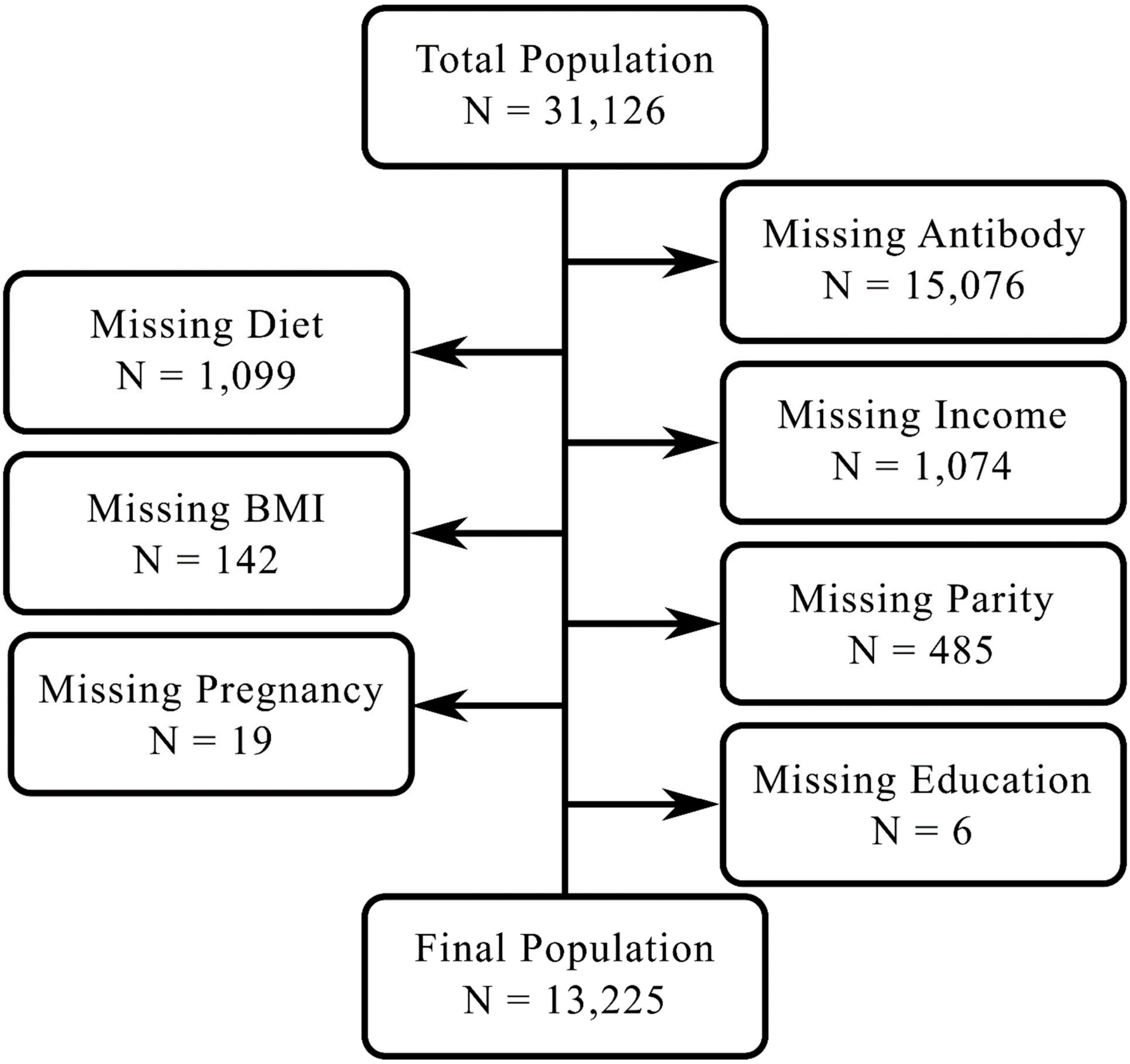

SM Figure 3: Reasons for exclusion from the main analysis (Mumps)

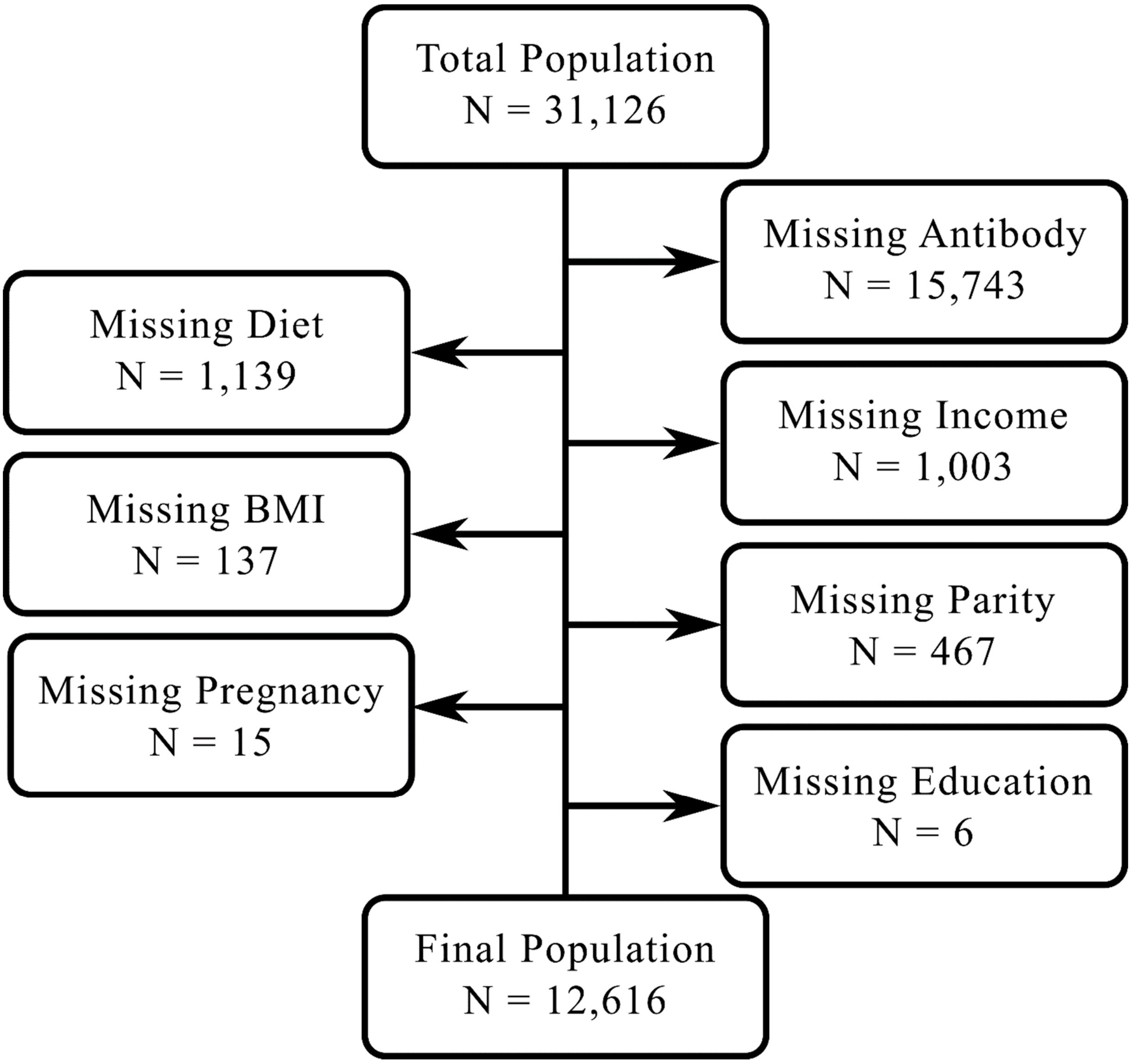

SM Table 1: Mean Dietary Fiber (g/d) Intake Contributed from Selected Food Sources Classified by Recoded What We Eat In America (WWEIA) Food Categories: NHANES, 1999-2004, Dietary Sample with Mumps Antibody Data (n = 12,616)*

Recoded WWEIA food category code	WWEIA Food Category Description	Fiber (g) Intake				
		Mean	±	SE	Total Pct (%)	Subtot Pct (%)
0	All Food Groups	15.15	±	0.22	100.00	
100	All Specific Food Groups Included in Analyses	12.05	±	0.18	79.55	100.00
1000	Fruit	1.22	±	0.05	8.06	10.13
2000	Vegetables	2.61	±	0.05	17.24	21.67
3000	Plant-based Protein Foods	1.33	±	0.06	8.76	11.01
3100	Beans, peas, legumes	0.84	±	0.06	5.56	6.99
3200	Nuts and seeds	0.43	±	0.03	2.83	3.55
3300	Processed soy products	0.06	±	0.02	0.38	0.47
4000	Grain Foods, Grain-Based Mixed Dishes, Pizza and Sandwiches	5.13	±	0.10	33.84	42.54
4100	Grain Foods	3.40	±	0.09	22.42	28.18
4200	Grain-based Mixed Dishes, Pizza and Sandwiches	1.73	±	0.03	11.42	14.35
4210	Grain-based Mixed Dishes	0.82	±	0.04	5.43	6.83
4220	Pizza	0.61	±	0.02	4.03	5.07
4230	Sandwiches (single code)	0.30	±	0.01	1.95	2.46

5000	Savory Snacks, Crackers, Snack/meal Bars, and Sweet Baked Goods	1.77	±	0.03	11.65	14.65
5100	Savory Snacks and Crackers	1.08	±	0.03	7.14	8.97
5200	Snack/Meal Bars and Sweet Bakery Products	0.68	±	0.02	4.52	5.68
6000	Non-specific Food Groups Included in Analyses as One Group	3.10	±	0.09	20.45	
6100	Fruit and Vegetable Juices	0.18	±	0.01	1.19	
6110	Fruit Juices	0.16	±	0.01	1.05	
6120	Vegetable Juices	0.02	±	0.01	0.14	
6200	Mixed Dishes Not Included with Grain Foods	1.46	±	0.08	9.63	
6210	Mixed Dishes Mainly Meat, Poultry, or Seafood	0.46	±	0.03	3.05	
6220	Asian Mixed Dishes	0.18	±	0.02	1.18	
6230	Mexican Mixed Dishes	0.54	±	0.07	3.57	
6240	Soups	0.28	±	0.02	1.83	
6300	Protein/nutritional powders and foods not included in a category	0.05	±	0.02	0.31	
6400	Other Foods and Beverages	1.41	±	0.04	9.32	
6410	Animal-based Protein Foods	0.14	±	0.01	0.93	
6420	Milk and Dairy	0.22	±	0.01	1.47	
6430	Nonalcoholic Beverages (excluding 100% Juice), Alcoholic Beverages, and Water	0.19	±	0.01	1.25	
6440	Fats and Oils, Condiments and Sauces, Sugars, Infant Formula and Baby Food	0.47	±	0.02	3.09	
6450	Candy and Other Desserts	0.39	±	0.02	2.58	

*Sample-weighted mean and standard error are estimated using SUDAAN.

The food groups used in the analysis (n = 6) are in column two. The subgroups of those shown in columns 3 and 4 are for explanatory reasons only.

SM Table 2. Characteristics of NHANES subjects 1999-2004 included in the main analyses[a]

Characteristic	Median (and quartiles), or percent (n = 12616)
Age	28 (16, 38)
Sex	
Female	48.0
Male	52.0
Race/Ethnicity	
Mexican American	10.2
Other Hispanic	5.9
Non-Hispanic White	66.6
Non-Hispanic Black	12.1
Other Race	5.2
Education	
< 9th grade	24.5
Grades 9 to 11	15.4
High School or GED (includes those in Grade 12)	19.3
Some College	23.4
College	17.4
Income-Poverty Ratio	2.6 (1.2, 4.5)
Survey Year	
1999-2000	27.8
2001-2002	35.6
2003-2004	36.6
BMI (kg/m^2)	24.8 (20.8, 29.5)
0 children	41.5
1 child	15.6
2 or more	42.9
Pregnant (females, ages 12 - 49) [b]	4.8
Breastfeeding (females, ages 12 - 49) [b]	2.6
Smoking (ages 12 - 49) [c]	
Never [<100 lifetime cigarettes]	57.5
Former [not current smoker]	17.4
Smoker [< 1 pack per day]	15.2
Heavy Smoker [≥ 1 pack per day]	9.9

(cont.)

(cont.)

Alcohol Use (ages 20 – 49) [d]	
Never [<12 lifetime drinks]	11.5
Former [0 drinks last 12 months]	1.9
Light Drinker [<1 drink per week]	46.7
Drinker [<7 drinks per week]	36.7
Heavy Drinker [≥7 drinks per week]	3.2
Dietary intake	
Crude Dietary Fiber (g/day)	13.3 (8.9, 19.3)
Energy Adjusted Fiber (g/day)	13.8 (10.7, 18.1)
Energy Adjusted Fiber (g/day) /IUR	1.8 (1.4, 2.4)
Total Energy Intake (kcal/day)	2,164 (1,634, 2,831)
Vitamin C (mg)	60.7 (27.8, 123.5)
Vitamin E (mg)	6.3 (4.2, 9.2)
Carotene (mcg RE)	774 (330, 2,269)
Protein (gm)	77.0 (55.6, 104.0)
Selenium (mcg)	98.4 (70.4, 137.1)
Zinc (mg)	10.8 (7.5, 15.7)
Vitamin B6 (mg)	1.7 (1.1, 2.4)
Folate (mcg)	360.7 (247.8, 512.8)
Magnesium (mg)	250.4 (179.0, 343.4)
Copper (mg)	1.1 (0.8, 1.5)
Vitamin A (mcg)	520.0 (292.9, 853.6)
Supplements	
Crude Supplement Fiber (g/day)	0.0 (0.0, 0.0)
Vitamin C (mg)	0.0 (0.0, 47.2)
Vitamin E (mg)	0.0 (0.0, 7.2)
Carotene (mg)	0.0 (0.0, 0.0)
Protein (gm)	0.0 (0.0, 0.0)
Selenium (mcg)	0.0 (0.0, 0.0)
Zinc (mg)	0.0 (0.0, 0.5)
Vitamin B6 (mg)	0.0 (0.0, 0.8)
Folate (mcg)	0.0 (0.0, 66.7)
Magnesium (mg)	0.0 (0.0, 0.0)
Copper (mg)	0.0 (0.0, 0.0)
Vitamin A (mcg)	0.0 (0.0, 206.6)

(cont.)

(cont.)

Met-Min/Month (ages 12 - 49) [e]	
< 2000	27.8
2000-3999	17.7
4000-5999	11.4
6000-7999	9.1
8000+	34.0
Antibody concentration (untransformed)	
Mumps	2.6 (1.7, 3.7)

[a] Values shown are for subjects with data on Mumps.

[b] Females 12-49, n = 5150

[c] With smoking data, n = 8977

[d] With alcohol data, n = 4415

[e] with Met-Min/Month data, n = 7608

SM Table 3. Age-adjusted median amount of energy-adjusted dietary fiber (g/d) according to category of subject characteristic (and quartiles) or Pearson correlation coefficient of energy-adjusted dietary fiber with continuous value of the subject characteristic

Characteristic	Median or r (n = 12616)[a]
Age	
6 to < 12 years	13.7 (11.4, 16.4)
12 to < 20 years	12.7 (10.2, 16.2)
20 - 49 years	14.3 (10.7, 19.2)
Sex	
Female	14.2 (11.4, 18.0)
Male	13.4 (10.0, 18.2)
Race/Ethnicity	
Mexican American	16.0 (12.3, 21.5)
Other Hispanic	14.0 (11.3, 18.2)
Non-Hispanic White	13.9 (10.7, 18.3)
Non-Hispanic Black	12.1 (9.6, 15.3)
Other Race	12.6 (9.6, 16.9)
Education	
< 9th grade	13.7 (11.2, 16.9)
Grades 9 to 11	12.5 (9.5, 16.4)
High School or GED (includes those in Grade 12)	12.8 (9.5, 16.8)
Some College	13.9 (10.9, 18.6)
College	16.7 (12.7, 22.7)
Income-Poverty Ratio	
1st Tertile	13.2 (10.2, 17.1)
2nd Tertile	13.4 (10.3, 17.5)
3rd Tertile	14.8 (11.4, 19.8)
Survey Year	
1999-2000	13.2 (10.0, 17.8)
2001-2002	13.8 (10.6, 18.4)
2003-2004	14.2 (11.3, 18.1)
BMI (kg/m^2)	-0.05
Parity (females, aged 12-49)[b]	
0 children	14.3 (11.4, 18.6)
1 child	14.0 (10.9, 18.3)
2 or more	14.2 (11.3, 18.6)
Pregnant (females, ages 12 - 49) [b]	
No	14.2 (11.3, 18.4)
Yes	15.0 (11.6, 19.6)

(cont.)

(cont.)

Breastfeeding (females, ages 12 - 49) [b]	
No	14.2 (11.3, 18.4)
Yes	15.5 (12.1, 21.7)
Smoking (ages 12 - 49) [c]	
Never [<100 lifetime cigarettes]	14.8 (11.2, 19.5)
Former [not current smoker]	15.0 (11.7, 20.6)
Smoker [< 1 pack per day]	12.0 (9.1, 15.8)
Heavy Smoker [≥ 1 pack per day]	10.8 (8.0, 14.2)
Alcohol Use (ages 20 - 49) [d]	
Never [<12 lifetime drinks]	15.4 (11.8, 20.5)
Former [0 drinks last 12 months]	13.5 (9.6, 19.3)
Light Drinker [<1 drink per week]	14.2 (10.8, 18.9)
Drinker [<7 drinks per week]	13.9 (10.0, 19.0)
Heavy Drinker [≥7 drinks per week]	13.4 (10.2, 20.5)
Dietary intake	
Vitamin C (mg)	0.27
Vitamin E (mg)	0.27
Carotene (mcg RE)	0.25
Protein (gm)	0.08
Selenium (mcg)	0.07
Zinc (mg)	0.14
Vitamin B6 (mg)	0.29
Folate (mcg)	0.43
Magnesium (mg)	0.67
Copper (mg)	0.34
Vitamin A (mcg)	0.17
Supplements	
Crude Supplement Fiber (g/day)	0.01
Vitamin C (mg)	-0.00
Vitamin E (mg)	-0.00
Carotene (mg)	0.04
Protein (gm)	0.06
Selenium (mcg)	0.04
Zinc (mg)	0.08
Vitamin B6 (mg)	0.05
Folate (mcg)	0.08
Magnesium (mg)	0.06
Copper (mg)	0.06
Vitamin A (mcg)	0.00

(cont.)

(cont.)

Met-Min/Month (ages 12 - 49) [e]	
< 2000	13.5 (10.3, 18.2)
2000-3999	14.7 (11.0, 18.9)
4000-5999	14.5 (11.4, 19.8)
6000-7999	14.1 (10.8, 19.0)
8000+	14.4 (10.7, 19.5)

[a] Results shown are for subjects with data on Mumps

[b] Females 12-49, n = 5150

[c] With smoking data, n = 8977

[d] with alcohol data, n = 4415

[e] with Met-Min/Month data, n = 7608

SM Table 4. Fully-adjusted percent difference in Mumps antibody concentration (%Δ) per interquartile range increment in dietary fiber intake (and 95% confidence interval), according to age group or tertile of income-to-poverty ratio[a]

Modifying Factor	%Δ	(95% CI)
Age		
< 12	5.18	(-11.49, 24.98)
12–19	8.32	(2.85, 14.07)
20+	6.55	(2.69, 10.55)
Income-to-poverty ratio (tertile)		
< 1.65	1.36	(-4.42, 7.48)
>=1.65 and < 3.86	6.30	(-2.31, 15.68)
>=3.86	10.70	(2.59, 19.47)

[a] Adjusted for all factors listed in Table 2. Mumps results are based on quadratic model for fiber.

SM Table 5. β coefficients from a fully-adjusted model of ln(Mumps antibody concentration) for fiber in g/d day from six groups of fiber-containing foods (n = 12,616). Results are adjusted for energy intake.

Food group	β	Standard Error of β
Fruits	0.0073	0.0047
Vegetables	0.0038	0.0031
Plant-based Protein Foods	0.0023	0.0021
Grain Foods, Grain-Based Mixed Dishes, Pizza and Sandwiches	0.0085	0.0021
Savory Snacks, Crackers, Snack/meal Bars, and Sweet Baked Goods	-0.0086	0.0035
All other foods	0.0011	0.0026

F-test for improvement in model fit as compared with that used for Mumps in Table 3, $p < 0.000001$

SM Table 6. Results from multiple imputation analysis. Fully-adjusted percent difference (%Δ) in antibody concentration per interquartile range increment in energy-adjusted fiber (and 95% confidence interval).[a]

Antibody type	%Δ	(95% CI)
Measles	0.37	(-4.15, 5.10)
Mumps	5.93	(2.91, 9.04)
Rubella	0.99	(-3.87, 6.09)
Varicella	-2.46	(-6.06, 1.28)

[a] Adjusted for all the factors listed in Table 2. Mumps results are based on quadratic model for fiber. The number of subjects for the analyses of Measles, Rubella, and Varicella was 14951; for Mumps it was 14244.

SM Table 7. Results after excluding subjects who had medical conditions or therapeutic drug use (past 30 days) associated with secondary antibody deficiency. Fully-adjusted percent difference (%Δ) in antibody concentration per interquartile range increment in energy-adjusted fiber (and 95% confidence interval).[a]

Antibody type	%Δ	(95% CI)
Measles	0.32	(-4.72, 5.63)
Mumps	5.31	(0.80, 10.02)
Rubella	0.65	(-4.71, 6.32)
Varicella	-2.93	(-6.63, 0.92)

[a] Adjusted for all the factors listed in Table 3. Mumps results are based on quadratic model for fiber. The number of subjects in the analyses for Measles, Rubella, and Varicella was 12721; for Mumps it was 12153.
